# Exosomes in lung cancer: a role in early diagnosis

**DOI:** 10.3389/fonc.2025.1599608

**Published:** 2025-06-12

**Authors:** Tong Zhou, Hui Ma, Zhikang Li, Yijun Xu, Lingling Zhao

**Affiliations:** ^1^ Wuhan Kindstar Zhenyuan Medical Laboratory Co., Ltd., Wuhan, China; ^2^ Chest Hospital, Tianjin University, Tianjin, China; ^3^ Department of Thoracic Surgery, Tianjin Chest Hospital, Tianjin, China; ^4^ Department of Scientific Research, Kindstar Global Precision Medicine Institute, Wuhan, China

**Keywords:** lung cancer, exosomes, early diagnosis, biomarkers, liquid biopsy

## Abstract

Lung cancer is the most prevalent and deadly malignant tumor in the world. Traditional treatment methods rely on histopathological analysis of cancer cells obtained through tissue biopsies, which carry risks due to their invasive nature. Thus, there is an urgent need to identify effective and non-invasive early screening methods for lung cancer. Exosomes, a crucial element of liquid biopsies, have emerged as a promising alternative due to their non-invasive collection, convenience and cost-effectiveness in diagnosing lung cancer. Research has underscored the role of exosomes in lung cancer invasion, metastasis, immune regulation, and the tumor microenvironment. Furthermore, the contents of exosomes, such as miRNAs, lncRNAs, circRNAs, and proteins, demonstrate considerable potential for the early diagnosis of lung cancer. This article provides a comprehensive review of the role and application of exosomes as liquid biopsy markers for early diagnosis of lung cancer, emphasizing their promise in improving patient outcomes through earlier detection and intervention.

## 1 Background

**Figure 1:**
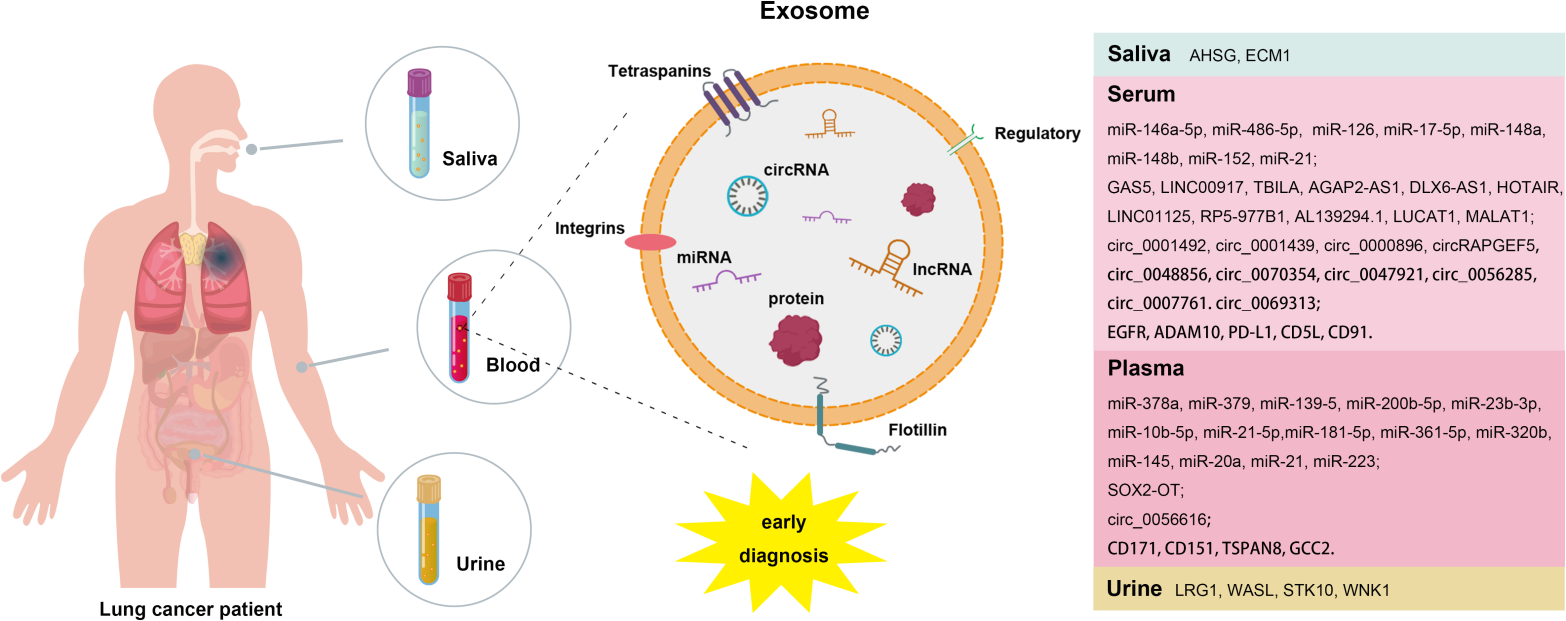
Exosome-related biomarkers for early diagnosis of lung cancer.

Lung cancer is a malignant tumor with a rapidly increasing incidence and mortality rare worldwide, representing a significant threat to human health. It encompasses various subtypes, including small cell lung cancer (SCLC), lung adenocarcinoma (LUAD), Lung squamous cell carcinoma (LUSC), and large-cell carcinoma (LCC) (1, 2). Currently, surgical resection remains the most effective treatment for early-stage lung cancer, however, the early diagnosis rate is only 15%, with fewer than 30% of patients meeting clear surgical criteria (3–6). Early screening is crucial for reducing mortality from non-small cell lung cancer (NSCLC) (7). Conventional clinical tests, such as magnetic resonance imaging (MRI) and computed tomography (CT), are often costly and can lead to false positives. While tumor markers can provide additional risk assessment for clinical decision making (8, 9), pathological biopsy, considered the gold standard for lung cancer diagnosis, is not suitable for early screening and follow-up due to its invasive nature. Liquid biopsy has emerged as a promising diagnostic technique characterized by its non-invasive approach, high specificity, sensitivity and early detection capabilities, with exosomes gaining attention as potential biomarkers for this method (10, 11). Exosomes are small lipid bilayer vesicles that contain a wealth of nucleic acids, proteins, and lipids, thereby facilitating liquid biopsy (12). Their stability is a key advantage, as the lipid bilayer structure protects their contents from degradation. Moreover, exosomes carry a complete set of genetic information from their parental cells, making them effective biomarkers that accurately reflect the physiological state of those cells. Notably, exosomes derived from tumor cells are enriched with tumor-specific DNA, RNA, and proteins (13–16). Recent studies have underscored the strong connection between exosomes and lung cancer, influencing various aspects of tumor progression, including proliferation, metastasis, drug resistance and angiogenesis. Consequently, exosomes are positioned as ideal biomarkers for the early diagnosis of lung cancer (17–21). This article provides an overview of research findings on exosomal miRNAs, lncRNAs, circRNAs and proteins as biomarkers, offering new insights for the early diagnosis and treatment of lung cancer (**Figure 1**).

## 2 Overview of exosomes

### 2.1 Biological characteristics of exosomes

The study of exosomes began in 1987, when Johnstone et al. (22) first observed that reticulocytes release vesicles into the extracellular space, coining the term “exosomes” based on their morphology and size. Exosomes are small vesicles with a diameter of 30-100nm and have a disc-like structure characterized by a bilayer lipid membrane (23). They are enriched proteins related to multivesicular body (MVB), including Flotillins, Annexins, GTPases, RABs, SNAREs, ALIX, TSG101, heat shock proteins (HSP70, HSP90) and transmembrane protein families such as CD9, CD63, CD81 and CD82 (23). These proteins play crucial roles in various biological processes of exosomes, including biogenesis, antigen presentation, membrane transport, and fusion (24, 25). In addition to proteins, exosomes contain a diverse array of nucleic acids, such as mRNAs, miRNAs, lncRNAs, circRNAs and snoRNAs (26–28). They also encompass various lipid components, including cholesterol (CHOL), sphingomyelin (SM), phosphatidylcholine (PC), phosphatidylserine (PS), phosphatidylethanolamine (PE), diacylglycerol (DAG), phosphatidic acid (PA), and phosphatidylinositol (PI) (29). These components contribute to the functional characteristics of exosomes, mirroring those of the parental cells and highlighting their potential as biomarkers (30–32).

### 2.2 Biogenesis of exosomes

Currently, the biogenesis of exosomes is primarily understood to occur through two pathways: the plasma membrane and endosome. The endosomal pathway, which has been extensively studies, involves the formation of early endosomes from the plasma membrane, the generation of MVBs, and their subsequent fusion with the plasma membrane to release exosomes (22). Additionally, exosomes can bud directly from the plasma membrane, a process referred to as the plasma membrane pathway. MVB formation involves two main mechanisms: ESCRT-dependent and ESCRT-independent mechanisms. The endosomal sorting complex required for transport (ESCRT) comprises four protein complexes, along with accessory proteins (ESCRT-0, ESCRT-I, ESCRT-II, ESCRT-III) (32). ESCRT plays a crucial role in MVB production by facilitating cargo aggregation, membrane invagination, and vesicle neck division (33). For instance, CD63 is directly involved in the ESCRT-independent sorting of premelanosome protein (PMEL) in human melanoma cells. In HEK293, CD82 and CD9 promote the excretion of exosomes enriched with β-catenin (34). Similarly, the tetraspanin Tspan8 is responsible for recruiting specific proteins and mRNAs to exosomes in pancreatic cancer (35). Notably, inhibiting these pathway dose not eliminate exosome production (36), suggesting that cells can utilize multiple pathways for MVB formation. Furthermore, the same MVB may arise from intracellular vesicles formed through different mechanisms.

The rate of exosome synthesis and secretion varies significantly among different cell types (37). For example, tumor cells exhibit increased exosome secretion during radiotherapy (38), leading to higher levels of exosomes in the blood of cancer patients compared to healthy individuals (39). Although the precise mechanisms governing exosome function are not fully understood, substantial research has explored their roles. Initially, exosomes were considered as cellular “garbage bag” responsible for exporting excess or non-functional components from cells (40). However, recent studies have revealed that exosomes originate from diverse sources and possess distinct biological functions. They play important roles in various physiological processes, including immune surveillance, neuroshaping, tissue repair, stem cell survival, and coagulation (41). Moreover, exosomes hold an irreplaceable role in pathological conditions, particularly in tumors development (30).

### 2.3 Extraction of exosomes

High purity of exosomes is essential for effective exosome research. Various extraction methods have been developed based on the characteristics of exosomes, such as size, density, and surface markers (42). Among these, ultracentrifugation is the most commonly used technique. This method first eliminates cell debris through low- and medium-speed centrifugation, followed by ultracentrifugation to separate and concentrate the exosomes. It is considered the gold standard due to its low cost, minimal risk of contamination, and high yield (43, 44). However, it requires a large sample volume and often results in lower purity of the isolated exosomes. Density gradient centrifugation is another method that utilizes biocompatible media, such as sucrose, to separate particles based on their different densities (45, 46). This approach can achieve higher purity of exosomes but is not suitable for small-volume samples. The immunocapture method (47) involves isolating and enriching exosomes by capturing their surface markers with specific antibodies. While this technique does not compromise the morphology of exosomes, it comes with high reagent costs and a reliance on the availability of specific antibodies.

In addition to these traditional methods, microfluidic technology has emerged as a promising approach for efficient exosome isolation from biological fluids. For instance, the automated centrifugal microfluidic disk system combined with functionalized membranes (Exo-CMDS) achieved a diagnostic accuracy of 91% for lung cancer detection in trace blood samples (15). Although microfluidic technology is user-friendly and offers a high capture rate, it typically requires integration with other instruments, and relying on a single separation method may impact both the purity and recovery of exosomes. Other extraction techniques, such as size exclusion chromatography (48), ultrafiltration (49), and polymer precipitation (50), also exist, each with its own advantages and disadvantages. No single method has yet proven capable of efficiently extracting exosomes from all types of samples. In summary, the extraction of exosomes continues to face significant challenges and requires further research and optimization to enhance its efficiency and purity.

## 3 Role of exosomes in lung cancer development

Immunosuppression: Tumor-derived exosomes actively interact with immune cells, transmitting inhibitory signals that result in a reduced number of antigen-presenting cells (APCs) and the suppression of T cell and natural killer (NK) cell activity. This interaction allows cancer cells to evade immune surveillance, induces immune tolerance, and further facilitates the growth, metastasis and invasion of tumor cells (51–57).

Proliferation: Platelet exosomes isolated from lung cancer patients have been observed to transfer CD41 to the surface of lung cancer cells. This transfer induces the expression of cyclin D2 in these cells, leading to the upregulation of MAPKp42/44 phosphorylation, which subsequently promotes lung cancer cell proliferation (58). Additionally, KLF9, a crucial factor for cell proliferation, differentiation, and tissue development, is targeted by plasma exosomal miR-660-5p, facilitating the progression of NSCLC (59). Furthermore, exosomal miR-96 has been shown to enhance the proliferation of LUAD H1299 cells by targeting LMO7 expression (60). Exosomal miR-29a and miR-21, derived from A549 cells, can bind to toll-like receptors (TLRs) on immune cells within the tumor microenvironment, thereby influencing lung cancer cell proliferation (61).

Metastasis: Metastasis is a complex process that involves the invasion, survival, attachment, and colonization of tumor cells in distant organs. Exosomes serve as key mediators of intercellular communication and play a crucial role in various stages of metastasis. Firstly, exosomes promote epithelial-mesenchymal transition (EMT), a phenomenon strongly linked to lung cancer metastasis. Specific exosomal miRNAs such as miR-193a-3p, miR-210-3p and miR-5100, active STAT3 signaling to induce EMT, thereby facilitating invasion and metastasis. Additionally, miR-23a regulates E-cadherin expression, maintaining EMT through the TGF-β pathway (62, 63). Secondly, exosomes stimulate angiogenesis by acting as messengers between tumor cells and vascular endothelial cells. For instance, overexpression of miR-210 in exosomes derived lung cancer patients activates the JAK2/STAT3 pathway, resulting in increased expression of pro-angiogenic factors such as MMP9, FGF2, and vascular endothelial growth factor (VEGF) (64). Abnormal expression of miRNA-497, miR-21 and miR-549a in exosomes from lung cancer cells has also been associated with angiogenesis (65–68). Lastly, exosomal RNA from mouse lung cancer cells has been shown to upregulate the expression of toll-like receptor 3 (TLR3) in type II alveolar epithelial cells through the NF-κB and MAPK pathways. This upregulation promotes the production of chemokines and neutrophil aggregation, ultimately contributing to the formation of a premetastatic niche (69).

## 4 Exosomes as biomarker for early diagnosis of lung cancer

### 4.1 Exosomal miRNAs

Due to their stability, accessibility and specificity, miRNAs encapsulated in exosomes are considered highly promising biomarkers for the early diagnosis of lung cancer. Numerous studies have identified specific miRNAs in exosomes that show diagnostic value for early-stage lung cancer. For instance, the study reported that the expression levels of four serum miRNAs (miR-21-5p, miR-1413p, miR-222-3p and miR-486-5p) and two serum exosomal miRNAs (miR-146a-5p and miR-486-5p) in patients with early-stage NSCLC were significant differences compared to those in patients with benign lung lesions and healthy individuals (70). The findings indicate that the area under the receiver operating characteristic curve (AUC) for these four serum miRNAs and the two serum exosomal miRNAs in early-stage NSCLC patients was ≥ 0.697, with the AUC for serum exosomal miRNAs exceeding that of serum miRNAs. This suggests that exosomal miRNAs hold considerable promise for application in the early diagnosis of lung cancer. Additionally, Jin et al. (71) conducted an analysis of plasma exosomal miRNAs from patients with early-stage NSCLC using miRNA sequencing and validated their results through qPCR. Their study identified two miRNAs (miR-181b-5p and miR-361-5p) specific to LUAD that were upregulated compared to healthy individuals and two that were downregulated (miR-30a-3p and miR-30e-3p). Furthermore, they also discovered three microRNAs specific to LUSC: miR-320b was found to be upregulated, while miR-10b-5p and miR-15b-5p were downregulated. The researchers also detected the expression levels of miR-378a, miR-379, miR-139-5p, miR-200b-5p, miR-19-3p, miR-21-5p, and miR-221-3p in the plasma exosomes, and miR-126, miR-17-5p, miR-21, miR-25, and miR-223 in serum exosomes of LUAD patients were significantly different from those in healthy individuals (72–79). Another study demonstrated that exosomes contribute to tumor growth and metastasis by delivering miR-1228-5p, positioning it as a potential biomarker for the diagnosis and prognosis of SCLC (80, 81). Therefore, exosomal miRNAs offer unique advantages and significant potential for the early diagnosis of lung cancer. More detailed information can be found in **Table 1**.

**Table 1:** Exosomal miRNAs involved in the early diagnosis of lung cancer.

Exo-miRNA	Source	Diagnostic value	Reference
miR-146a-5p, miR-486-5p	serum	The AUC of the combination in the early diagnosis of lung cancer was 0.898.	(70)
miR-378a, miR-379, miR-139-5, miR-200b-5p	plasma	The AUC of the combination in distinguishing LUAD from lung granulomas was 0.760.	(78)
miR-23b-3p, miR-10b-5p, miR-21-5p	plasma	The AUC for diagnosing NSCLC, when combined with clinical variables, was 0.91.	(81)
miR-181-5p, miR-361-5p, miR-320b, miR-10b-5p	plasma	The AUC of the combination in distinguishing NSCLC patients from non-NSCLC individuals was 0.899, with a sensitivity of 80.25% and a specificity of 92.31%. For diagnosing LUAD, the AUC was 0.936, with a sensitivity of 80.65% and a specificity of 91.67%. For diagnosing LUSC, the AUC was 0.911, with a sensitivity of 83.33% and a specificity of 90.32%.	(71)
miR-126	serum	The AUC for the early diagnosis of NSCLC was 0.875.	(72)
miR-17-5p	serum	The AUC for the early diagnosis of NSCLC was 0.746, with a sensitivity of 70.0% and a specificity of 82.2%.	(73)
miR-145, miR-20a, miR-21, miR-223	plasma	The AUC for the combination in the early diagnosis of NSCLC was 0.897, with a sensitivity of 81.8% and a specificity of 90.1%.	(75)
miR-148a, miR-148b, miR-152, miR-21	serum	The AUC for the combination in the early diagnosis of NSCLC was 0.98, with a sensitivity of 96% and a specificity of 91%.	(76)

### 4.2 Exosomal lncRNAs

Long non-coding RNA (lncRNA), defined as RNA molecules longer than 200 nucleotides that do no encode proteins, play crucial roles in various important biological processes, including X chromosome silencing, chromatin modification, transcriptional activation and nuclear transport. They are closely associated with the occurrence, development and drug resistance of lung cancer (82–84). Recent studies have indicated that exosomal lncRNAs have promising potential as biomarkers for the early diagnosis of lung cancer. For example, the levels of serum exosomal lncRNA GAS5 in patients with early-stage NSCLC were found to be lower than those in healthy individuals, and with even lower levels observed in patients with advanced NSCLC. The AUC, sensitivity and specificity of exosomal lncRNA GAS5 for the diagnosis of NSCLC were 0.857, 85.94% and 70.00%, respectively. When combined with carcinoembryonic antigen (CEA), a commonly used nonspecific serum tumor biomarker for NSCLC, the AUC increased to 0.929 (85, 86). Additionally, another study revealed that serum exosome LINC00917 was expressed at higher levels in lung cancer patients compared to healthy controls, demonstrating significant diagnostic value for both early-stage and advanced lung cancer (87). Tao et al. (88) reported elevated expression of TGF-β induced lncRNA (TBILA) and AGAP2 antisense RNA 1 (AGAP2-AS1) in the serum exosomes of lung cancer patients when compared to healthy individuals. TBILA showed notable discriminatory capacity for all NSCLC patients, stage I NSCLC patients and adenocarcinoma (ADC) patients, while AGAP2-AS1 exhibited a higher AUC in distinguishing LUSC patients from healthy controls. Another study demonstrated significant up-regulation of exosomal lncRNA SOX2-OT in LUSC patients, with an AUC of 0.815, sensitivity of 76.00% and specificity of 73.17% for LUSC diagnosis (89). As research continues to advance, various exosomal lncRNAs, including growth-arrest specific protein 6 antisense RNA 1 (DLX6-AS1) (90), HOX transcript antisense RNA (HOTAIR) (91), LINC01125 (92), RP5-977B1 (93) (84), AL139294.1 (94), LUCAT1 (95, 96) and MALAT1 (97), have been identified as having clinical value in the diagnosis of lung cancer. More detailed information can be found in **Table 2**.

**Table 2:** Exosomal lncRNAs involved in the early diagnosis of lung cancer.

Exo-lncRNA	Source	Diagnostic value	Reference
GAS5	serum	The AUC for diagnosing NSCLC was 0.857, with a sensitivity of 85.94% and a specificity of 70.00%.	(85)
LINC00917	serum	The AUC was 0.811 for all NSCLC patients, 0.773 for I/II NSCLC, and 0.907 for III/IV NSCLC.	(87)
TBILA	serum	The AUC for diagnosing NSCLC was 0.775, with a sensitivity of 64.7% and a specificity of 80.7%.	(88)
AGAP2-AS1	serum	The AUC for diagnosing was 0.784, with a sensitivity of 75.0% and a specificity of 73.3%.	(88)
SOX2-OT	plasma	The AUC for diagnosing LUSC was 0.815, with a sensitivity of 76.0% and a specificity of 73.27%.	(89)
DLX6-AS1	serum	The AUC for diagnosing NSCLC was 0.806, with a sensitivity of 77.5% and a specificity of 85.9%.	(90)
HOTAIR	serum	The AUC for diagnosing NSCLC was 0.821, with a sensitivity of 88.9% and a specificity of 78.3%.	(91)
LINC01125	serum	The AUC for distinguishing early-stage NSCLC patients from disease-free controls was 0.706, while it was 0.666 for distinguishing them from and tuberculosis controls.	(92)
RP5-977B1	serum	The AUC for distinguishing early-stage NSCLC patients from healthy individuals was 0.8658.	(93)
AL139294.1	serum	The AUC for diagnosing NSCLC was 0.915.	(94)
LUCAT1	serum	The AUC for diagnosing LUAD was 0.852, with a sensitivity of 85.45% and a specificity of 77.38%.	(95)
MALAT1	serum	The AUC for diagnosing NSCLC was 0.80, with a sensitivity of 68% and a specificity of 95%.	(97)

### 4.3 Exosomal circRNAs

Exosomes-derived circular RNAs (circRNAs) have emerged as a new focus in non-coding RNA research, following the discoveries of miRNAs and lncRNAs. They hold significant promise as reliable biomarkers for the early diagnosis of lung cancer, potentially outperforming traditional tumor markers and serum circRNA. Analysis of serum exosomal circRNAs expression levels in LUAD patients before and after surgery revealed significant decrease in the expression of exosomal circ_0001492, circ_0001439 and circ_0000896 following the surgical procedure. ROC curve analysis demonstrated that the AUCs for these exosomal circRNAs were all greater than 0.75, and their combined diagnostic performance demonstrated higher sensitivity and specificity, with an AUC of 0.805 (98). Additionally, a study found that exosomal circRAPGEF5 exhibited strong diagnostic capability for LUAD, with an AUC, sensitivity and specificity of 0.847, 64.90% and 95.60%, respectively, and the combination of circRAPGEF5 with CEA further improved diagnostic efficacy (99). Furthermore, He et al. (100) identified exosomal circRNA_0056616 as being closely associated with lymph node and distant metastasis in LUAD, demonstrating high sensitivity and specificity for diagnosing lymph node metastasis, with an AUC of 0.812. Several other exosomal circRNAs have also demonstrated significant diagnostic value for NSCLC, including circ_0048856 (101), hsa_circ_0070354 (102), circ_0047921, circ_0056285, circ_0007761 (103), circ_0069313 (104) and circFARSA (105). More detailed information about these exosomal circRNAs can be found in **Table 3**. In summary, exosomal circRNAs has high specificity and sensitivity as biomarkers for the early diagnosis of lung cancer, potentially surpassing traditional diagnostic markers. The combined use of multiple exosomal circRNAs may provide greater specificity and sensitivity compared to individual exosomal circRNA.

**Table 3:** Exosomal circRNAs involved in the early diagnosis of lung cancer.

Exo-circRNA	Source	Diagnostic value	Reference
circ_0001492	serum	The AUC for diagnosing NSCLC was 0.929.	(98)
circ_0001439	serum	The AUC for diagnosing NSCLC was 0.829.	(98)
circ_0000896	serum	The AUC for diagnosis of NSCLC was 0.880.	(98)
circRAPGEF5	serum	The AUC for distinguishing LUAD patients from healthy individuals was 0.847, with a sensitivity of 64.90% and a specificity of 95.60%.	(99)
circ_0056616	plasma	The AUC for diagnosing lymph node metastasis in LUAD was 0.812, with a sensitivity of 79.2% and a specificity of 81.0%.	(100)
circ_0048856	serum	The AUC for diagnosing NSCLC was 0.943, with a sensitivity of 88.0% and a specificity of 80.0%.	(101)
circ_0070354	serum	The single diagnosis of NSCLC, the AUC was 0.66, with a sensitivity of 52.63% and a specificity of 76.29%. The combined diagnosis using CEA, SCC, Cyfra21–1 and circ_0070354, the AUC was 0.73, with a sensitivity of 63.91% and a specificity of 84.54%.	(102)
circ_0047921, circ_0056285, circ_0007761	serum	The AUC for the combination in distinguishing NSCLC patients from healthy individuals was 0.926.	(103)
circ_0069313	serum	The AUC for distinguishing NSCLC from benign lung tumor was 0.749.	(104)

CEA, SCC and Cyfra21–1 are mature biomarkers of lung cancer.

### 4.4 Exosomal proteins

Exosomes contain a rich variety of proteins derived from their parental cells, making the detection of these exosomal proteins a valuable diagnostic approach for lung cancer (106). For example, epidermal growth factor receptor (EGFR), a crucial regulator of tumor growth, can be detected in the plasma of patients with early-stage lung cancer through exosomes (107). Approximately 80% of exosomes isolated from lung cancer samples contain EGFR, in stark contrast to only about 2% of exosomes from samples of chronic lung inflammation (108). While the levels of exosomal EGFR is significantly increased in lung cancer patients, the concentration of soluble EGFR in plasma does not show a notable difference (109). Moreover, Yoneyama et al. (110) found that active disintegrin and metalloproteinase domaincon-taining protein 10 (ADAM10) is significantly increased in the exosomes of NSCLC patients. This marker can effectively differentiate NSCLC patients from healthy individuals, establishing it as an important biomarker for NSCLC detection. Another important protein, programmed death-ligand 1 (PD-L1), has been identified in exosomes. PD-L1 binds to programmed death-1 (PD-1) on immune T cells and inhibit T-cell activation (111). The detection of PD-L1 in exosomes enables sampling from blood and other body fluids, providing an auxiliary diagnostic method for the early diagnosis of NSCLC (112). Studies have confirmed that exosomal PD-L1 levels in NSCLC patients are higher than those in normal controls, correlating with disease progression, clinicopathological characteristics, and TMN stage. Interestingly, no significant correlation has been found between PD-L1 levels in tumor tissue and the clinical characteristics of patients (113–115). Therefore, exosomal PD-L1 may offer greater clinical utility for diagnosing NSCLC compared to PD-L1 derived from tumor tissue. Choi et al. (116) demonstrated that the expression levels of seven proteins (CD5L, CLEC3B, ITIH4, SERFINF1, SAA4, SERFINC1 and C20-ORF3) were significantly increased in plasma exosomes of lung cancer patients. Further analysis revealed that only CD5L was significantly upregulated in cancer tissues, suggesting its potential as a biomarker for lung cancer diagnosis. Several studies have indicated that the expression levels of CD171, CD151, and TSPAN8 protein in plasma exosomes of lung cancer patients are significantly higher than healthy individuals, with CD151 and CD171 showing high expression in LUAD patients, while CD151 and TSPAN8 are elevated in LUSC patients (117). Ueda et al. (118) quantitatively identified CD91 as a LUAD-specific antigen on exosomes through a comprehensive analysis of 1369 exosome protein profiles, serving as a screening marker for lung cancer that is unaffected by gender or age. Compared to the CEA, exosomal CD91 exhibits improved detection sensitivity for early-stage lung cancer. High expression levels of GCC2 in the peripheral blood of patients demonstrate both specificity and sensitivity for early-stage lung cancer (119). Additionally, the proteins AHSG and ECM1 have been found to be significantly increased in the exosomes of NSCLC patients, suggesting their potential as diagnostic biomarkers for this condition (120).

In addition to exosomes derived from blood, several proteins have been reported to be significantly increased in the urine exosomes of NSCLC patients, including LRG1 (121), WASL, STK10 and WNK1 (122). One study identified twelve proteins, such as members of the annexin family (annexin A1, A2, A3, A5, A6, A11), along with PR-OM1, MUC1, BPIFA1, CRNN, MUC5B and IQGAP, which showed significant differences between lung cancer patients and healthy individuals. Furthermore, a comparative proteomics analysis revealed that MUC5B, IQGAP, ENO1 and SPARCL1 were identified in the salivary exosomes of lung cancer patients (123, 124). These exosomal proteins found in various body fluids show promise as potential biomarkers for lung cancer diagnosis. More detailed information can be found in **Table 4**. Given the diverse array of exosomal proteins, those sourced from different body fluids could serve as valuable biomarkers for lung cancer. It is also anticipated that combinations of various exosomal proteins may offer high clinical value as combined markers in the diagnosis and management of lung cancer.

**Table 4:** Exosomal proteins involved in the early diagnosis of lung cancer.

Exo-protein	Source	Diagnostic value	Reference
EGFR	serum	The level of exosomal EGFR in lung cancer patients was significantly increased.	(107–109)
ADAM10	serum	Distinguish NSCLC tumors from non-cancerous normal or lungs by COPD yield an AUC of 0.88, with sensitivity of 75.0% and specificity of 94%.	(110)
PD-L1	serum	A higher Exo-PD-L1 content was linked to larger tumor size, positive lymph node status, distant metastasis and advanced TNM stage in NSCLC patients.	(114)
CD5L	serum	CD5L, CLEC3B, ITIH4, SERFINF1, SAA4, SERFINC1 and C20ORF3 were all expressed at high levels in exosomes derived from lung cancer patients, with AUC > 0.750. Among these, CD5L exhibited the highest AUC of 0.943.	(116)
CD171, CD151, TSPAN8	plasma	The markers CD151, CD171, and TSPAN8 were the most effective in differentiating cancer patients of all histological subtypes from those without cancer, with CD151 showing AUC of 0.68 (p = 0.0002), CD171 showing AUC of 0.60 (p = 0.0002) and TSPAN8 showing AUC of 0.60 (p = 0.0002).	(117)
CD91	serum	The single diagnosis of NSCLC, the AUC was 0.724, with a sensitivity of 60.0% and specificity of 89.0%. The combined diagnosis using CEA yield an AUC of 0.882, along sensitivity of 71.4% and specificity of 91.8%.	(118)
GCC2	plasma	The AUC for diagnosing early-stage lung cancer was 0.844, with a sensitivity of 90.0% and a specificity of 75.0%.	(119)
AHSG, ECM1	saliva	The AUC for the combination in diagnosing early-stage NSCLC was 0.739, with a sensitivity of 87.5% and a specificity of 54.3%.	(120)
LRG1	urine	The expression was significantly increased, showing an approximately six-fold increase in the six NSCLC patients.	(121)
WASL, STK10, WNK1	urine	The AUC for the combination in diagnosing lung cancer was 0.760.	(122)

## 5 Conclusion and prospects

Currently, the diagnostic technology for lung cancer has seen significant advancements compared to the past. However, most existing methods primarily focus on detecting advanced lung cancer, leaving early diagnosis as a considerable challenge. Current diagnostic techniques, such as endoscopic ultrasound-guided fine needle aspiration (EUS-FNA), MRI, and computed tomography (CT) (125), often involve invasive procedures that carry risks for patients. While histopathological examination is widely regarded as the gold standard, its invasive nature cannot be overlooked. Given that several years may elapse between the onset of lung cancer and the appearance of symptoms, liquid biopsy has emerged as a vital tool for early screening (126). This method provides a promising opportunity to improve treatment outcomes and enhance survival rates for patients. Exosomes derived from tumor cells, which encapsulate a wealth of biological information, have shown considerable potential as biomarkers for the early diagnosis of lung cancer (127). However, the standardization of exosome isolation and detection technologies is crucial for their effective integration into early diagnosis protocols. Additionally, conducting more extensive clinical studies to validate the efficacy of exosomes as biomarkers is a necessary step for advancing this field.

Authoritative studies have highlighted the potential of exosomal miRNAs as biomarkers due to their essential role in regulating lung cancer proliferation and invasion. Nonetheless, there remains a significant gap in the widespread clinical application of miRNAs for lung cancer diagnosis. While specific diagnostic models incorporating multiple biomarkers have shown promising predictive performance, their low repeatability and complex composition present major challenges for the practical use of miRNAs in early detection (128). Advancements in sequencing technology have facilitated the identification of various lncRNAs and circRNAs that exhibit differential expression in exosomes. Accumulating evidence suggests that the expression of specific lncRNAs and circRNAs may provide valuable insights into the clinical features of lung cancer (129). Currently, the analysis of lncRNAs and circRNAs largely relies on bioinformatics predictions, underscoring the need for further experimental and clinical studies to validate their diagnostic value (130, 131). Furthermore, while previous research has shown exosomal proteins can be utilized for lung cancer diagnosis, the upregulation of these proteins may be influenced by various factors, including smoking and inflammation. Thus, diagnosing tumor-associated proteins in exosomes must be grounded in precise isolation and identification methods. It is essential to explore optimal diagnostic indicators through multidisciplinary research that encompasses clinical medicine, immunology, and bioinformatics, providing new directions and insights for the realization of personalized lung cancer diagnosis.

In summary, while research on early diagnosis of lung cancer faces numerous challenges, exosomes present significant promise as potential biomarkers. Future studies should emphasize technical standardization and clinical validation to enhance the practical application of exosomes in the early detection of lung cancer.
